# Formation of thyroid hormone revealed by a cryo-EM structure of native bovine thyroglobulin

**DOI:** 10.1038/s41467-022-30082-4

**Published:** 2022-05-02

**Authors:** Nils Marechal, Banyuhay P. Serrano, Xinyan Zhang, Charles J. Weitz

**Affiliations:** 1 Department of Neurobiology, Harvard Medical School, Boston, MA 02115 USA; 2 Institut de Génétique et de Biologie Moléculaire et Cellulaire, Illkirch, France

**Keywords:** Cryoelectron microscopy, Thyroid hormones

## Abstract

Thyroid hormones are essential regulators of metabolism, development, and growth. They are formed from pairs of iodinated tyrosine residues within the precursor thyroglobulin (TG), a 660-kDa homodimer of the thyroid gland, by an oxidative coupling reaction. Tyrosine pairs that give rise to thyroid hormones have been assigned within the structure of human TG, but the process of hormone formation is poorly understood. Here we report a ~3.3-Å cryo-EM structure of native bovine TG with nascent thyroid hormone formed at one of the predicted hormonogenic sites. Local structural rearrangements provide insight into mechanisms underlying thyroid hormone formation and stabilization.

## Abstract (web summary)

Thyroid hormones are formed within the precursor Thyroglobulin by a poorly understood process. Here Marechal et al. report a cryo-EM structure of native bovine Thyroglobulin with nascent thyroid hormone formed at a predicted hormone synthesis site.

## Introduction

Thyroid hormones T_3_ (tri-iodothyronine) and T_4_ (tetra-iodothyronine or thyroxine) are essential regulators of metabolism, development, and growth in all vertebrates and some invertebrates^1–3^. T_3_ and T_4_ are formed from the precursor thyroglobulin (TG), a ~660-kDa homodimer specific to the follicular lumen of the thyroid gland^1–3^. TG undergoes extensive tyrosine mono- and di-iodination, and a few pairs of the iodinated tyrosine residues become oxidatively coupled by a poorly understood reaction mechanism^3^. T_3_ is formed when the mono-iodinated phenolic ring of a donor tyrosine is transferred to a di-iodinated acceptor tyrosine; T_4_ is formed when the donor tyrosine phenolic ring is also di-iodinated^1–3^. This transfer produces an amino acid residue with a tri- or tetra-iodinated, double-ringed side chain at the acceptor tyrosine site (nascent thyroid hormone embedded within the TG polypeptide chain)^3^ and a residual dehydroalanine at the donor site^4,5^. In addition to being the site of hormone formation, TG functions as a reservoir for thyroid hormone storage until it is trafficked back into the thyrocyte and degraded in the lysosome, liberating T_3_ and T_4_ for secretion into the bloodstream^1–3^.

TG has four hormonogenic acceptor tyrosines (sites A-D) and five donor tyrosines, two for site A and one each for sites B-D^6^. In the three-dimensional structure of a pre-hormonogenic conformation of recombinant human TG, the principal factors determining which pairs of tyrosine residues can couple to form hormone appear to be exposure to solvent (presumably to allow iodination), flexibility of the local polypeptide chain, and a proximity within 15 Å^6^. Further progress in the mechanistic understanding of thyroid hormone synthesis, storage, and trafficking will require three-dimensional structures of TG subsequent to tyrosine coupling at the various hormonogenic sites. Here we report a ~3.3-Å cryo-EM structure of native bovine TG with nascent thyroid hormone present at the predicted hormonogenic site B.

## Results

### Preparation of TG and cryo-EM analysis

**Fig. 1:**
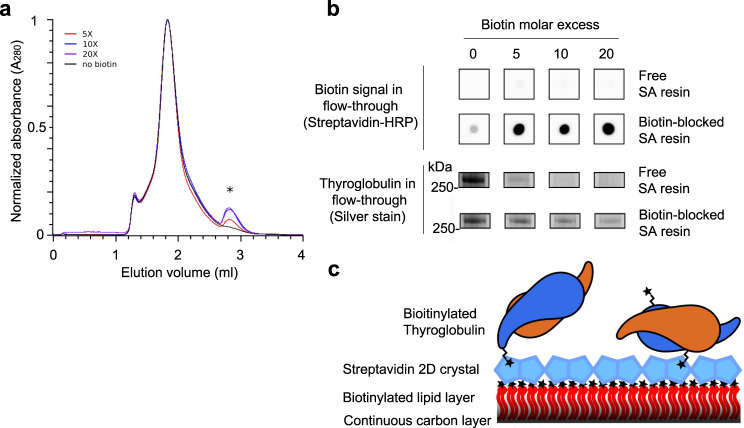
Biotinylation, purification, and capture of native bovine Thyroglobulin (TG) on streptavidin affinity cryo-EM grids. **a** Elution profile from preparative gel filtration chromatography (Superose 6 Increase GL resin) showing separation of biotinylated native TG from unreacted biotinylation reagents. Unbiotinylated control is plotted in black, and samples biotinylated with 5-, 10-, and 20-fold molar excess of biotin are plotted in the indicated colors. Asterisk, unreacted biotinylation reagents. **b** Analysis of TG biotinylation by adsorption to streptavidin (SA) resin. Top, streptavidin-HRP dot blots of SA resin flow-through fractions, as indicated. Bottom, silver-stained TG bands from SDS-polyacrylmide gel electrophoresis analysis of SA resin flow-through fractions, as indicated. Molecular weight marker running just below TG monomer is indicated at left. *N* = 3. Biotinylated TG from the 10-fold molar biotin reaction was selected for cryo-EM analysis on streptavidin affinity grids. Source data for TG SDS-PAGE analysis are provided as a Source Data file. **c** Schematic representation of randomly biotinylated homodimeric TG complexes (monomers are distinguished by blue and orange coloration) captured on a streptavidin affinity grid (streptavidin 2D crystal; pale blue; biotinylated lipids, red; continuous carbon, black). Stars represent biotins; tails protruding from the stars represent the flexible 60-Å linker.

**Fig. 2:**
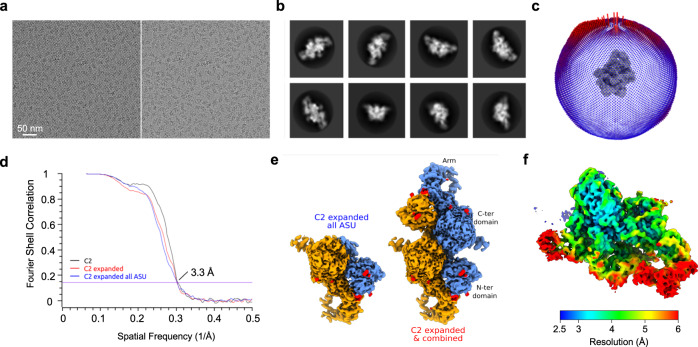
Cryo-EM analysis of native bovine TG. **a** Cryo-EM micrographs of biotinylated TG particles captured on a streptavidin affinity grid. Left, micrograph illustrating particles bound to the streptavidin 2D crystal lattice (5-nm array). Scale bar is at lower left. (*N* = 34,597). Right, the same image after subtraction of the lattice by Fourier filtering of Bragg spots^8^. **b** 2D class averages. **c** Angular distribution of TG particles. Blue-to-red color gradient marks under-to-over-represented orientations. **d** FSC curves showing the correlation between the indicated independent half-maps. **e** Reconstructions of native bovine TG from the indicated data processing procedures. Monomer subunits are colored blue and orange, respectively. N-linked glycosylation sites, red. **f** Unsharpened density map colored by local resolution (ranges from ~2.7 Å to 8.75 Å).

TG from bovine thyroid glands was lightly biotinylated and purified by gel filtration chromatography (Fig. 1a, b), after which it was captured on streptavidin affinity cryo-EM grids^7,8^ (Fig. 1c; Fig. 2a). The added biotin would be expected to pose negligible consequences for cryo-EM structure determination because the biotinylation reaction is not site-specific and the biotin moiety is separated from the protein by a 60-Å flexible linker (Fig. 1c). Streptavidin affinity grids protect proteins from the air-water interface during plunge-freezing, provide a favorable proteinaceous surface environment, facilitate diverse particle orientations, and allow concentration of complexes from solution onto the grid^8^.

**Fig. 3:**
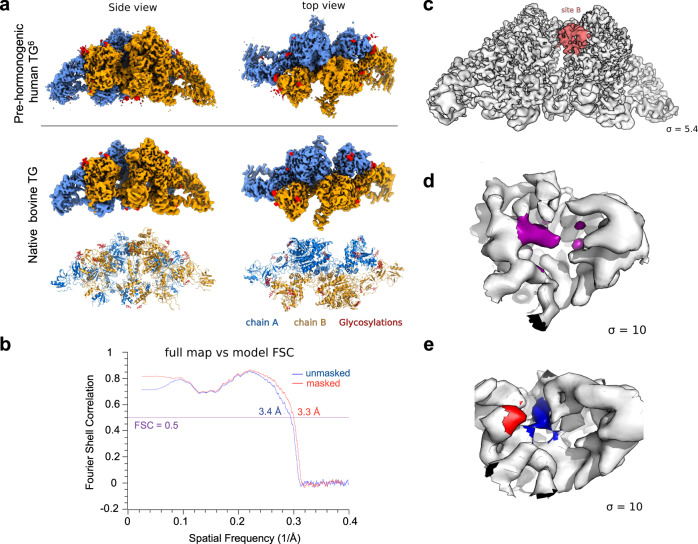
Global reconstruction of native bovine TG and initial density map for hormonogenic site B. **a** Upper and middle rows show, respectively, global reconstructions of pre-hormonogenic TG (from ref. ^6^) and the present analysis of native bovine TG (derived from the combined C2 map) color-coded as in Fig. 1; lower panel shows bovine TG reconstruction in ribbon representation. **b** FSC curves showing the correlation between the combined C2 density map and the atomic model. **c** C2 map of bovine TG showing the region encompassing hormonogenic site B (pale red). Sigma, contour level of map. **d** Detailed view of the density map for site B in native bovine TG. Novel unresolved density extending from the acceptor tyrosine position into an interior pocket is indicated in purple**. e** Corresponding view of the density map for site B in the pre-hormonogenic structure of human TG^6^. Acceptor and donor tyrosine densities are indicated in red and blue, respectively.

A cryo-EM reconstruction was obtained with a ~3.3 Å overall resolution and a ~2.8 Å local resolution over a substantial, presumably more rigid part of the complex (Fig. 2; Table 1), sufficient for building an atomic model. The structure is essentially identical to that of recombinant human TG^6^ (Fig. 3a), including conservation of multiple glycosylation sites (Fig. 3a; Supplementary Table 1). One notable difference was found in the region around hormonogenic site B (Y2573; numbered according to human TG in ref. ^6^), which exhibited unresolved extra density extending from the approximate position of the acceptor tyrosine residue toward the interior of the protein (Fig. 3b–d), suggesting the possibility of one or more structural variants superimposed in the map. We therefore performed 3D classification and local refinement (Supplementary Fig. 1) to resolve structural features at this site.

**Table 1:** Cryo-EM and model statistics.

Data collection and processing	Dimer	ASU
Microscope	FEI Krios	FEI Krios
Mode	EF-TEM Gatan	EF-TEM Gatan
Energy filter	Gatan GIF Quantum	Gatan GIF Quantum
Detector	K3	K3
Magnification	105 kX	105 kX
Voltage (kV)	300	300
Cumulative dose (e.Å^−2^)	54	54
Defocus range (μm)	0.5–3.5	0.5–3.5
Pixel size (Å)	0.825	0.825
Refinement symmetry	Combined C1 + C2	C1
Particles	242813	376342
Particles for C2-expanded	485626	
Map resolution (Å)	3.28	3.32
Map resolution range (Å)	2.71–10	2.79–8.75
Sharpening B factor (Å^2^)	−125.0	−92.0
Sharpening B factor (Å^2^) for C2-expanded	−101.6	
REFINEMENT		
PDB ID	7QTQ	
EMDB	EMD-14145	
Model composition:		
Non-hydrogen atoms	55253	
Protein residues	3577	
RMS deviations:		
Bond lengths (Å)	0.007	
Bond angles (°)	0.736	
Validation:		
Molprobity score (percentile)	2.08	
Clashscore	9.85	
Poor rotamers (%)	0	
Ramachandran plot:		
Favored (%)	89.29	
Allowed (%)	10.71	
Disallowed (%)	0

### Structure of bovine TG with nascent thyroid hormone

**Fig. 4:**
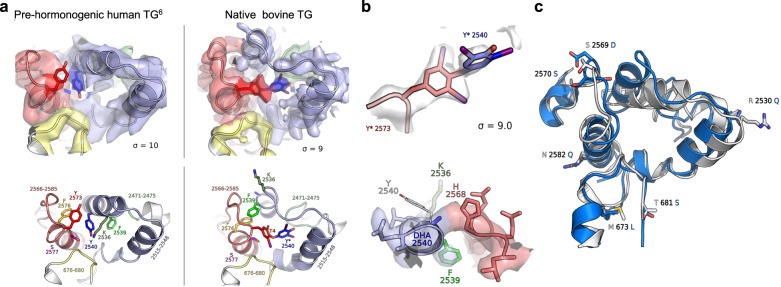
Nascent thyroid hormone at hormonogenic site B. **a** Left and right panels, respectively, show the pre-hormonogenic conformation of human TG (from Ref. ^6^) and the present analysis of native bovine TG. Top: Density maps of hormonogenic site B. Red, acceptor tyrosine Y2573; blue, donor tyrosine Y2540; green, F2539; gold, F2576; corresponding pale colors mark structural elements including the respective residues. Sigma, map contour level. Bottom: Atomic models derived from density maps in the top panel with the same color coding. The nascent thyroid hormone in the right panel is modeled as T_4_. Y*2540, transferred donor iodophenolic ring. Asterisk, residual donor site. **b** Top: Density map for the acceptor site superimposed on a model of T_4_ thyroid hormone (i.e., both aromatic rings modeled as 3,5-di-iodinated). Bottom: density map and model for the donor site showing fits to map of both the residual dehydroalanine (DHA 24540) and an unmodified Y2540. Maps of bovine TG in this figure were generated by density modification of cryoSPARC half-maps using the ResolveCryoEM tool from Phenix. **c** Overlay of hormonogenic site B regions of pre-hormonogenic human TG (white) and native bovine TG with nascent hormone (blue) illustrating the structural reorganization accompanying hormone formation. Positions of the non-conserved amino acid residues, all in peripheral locations, are marked.

This analysis succeeded in identifying a TG structure with nascent thyroid hormone present at hormonogenic site B (Fig. 4a). Close agreement between the density map and the model confirms that the acceptor tyrosine is 3,5-di-iodinated (Fig. 4b, top), as required for thyroid hormone formation. The map for the transferred donor phenolic group appears ambiguous regarding iodination state (Fig. 4b, top), as might be expected if the map includes a mixture of donor mono- and di-iodination states (corresponding to nascent T_3_ and T_4_, respectively), heterogeneity in the orientation of the donor aromatic ring, or both. Tandem mass spectrometry analysis (LC-MS/MS) confirmed that there was a mixture of nascent T3 and T4 at position 2573 in the TG sample (Supplementary Fig. 2).

At the predicted donor site (Fig. 4b, bottom), the map fits a dehyrdroalanine residue but does not appear to accommodate the original tyrosine aromatic side chain, whereas the map makes a good fit to other side chains of the loop containing the donor site (pale blue), including the aromatic side chain of the immediately adjacent residue F2439. Nearby H2568 is not well fit by the map, but it forms part of a separate loop (pale red) that makes a poorer fit overall. Although the result is somewhat ambiguous, the map is consistent with derivation of the donor phenolic ring at hormonogenic site B from Y2540, as concluded from biochemical experiments^6^.

We were unable to obtain a structure corresponding to a pre-hormonogenic state at site B, although we did detect the presence of unmodified Y2573 and di-iodinated Y2573 in the sample (Supplementary Fig. 2). Further 3D classification and focused refinement of hormonogenic sites A, C, and D did not result in maps with a resolution sufficient to determine the status of the assigned hormonogenic tyrosine pairs. The results suggest local heterogeneity, mobility, or both at these sites.

The nascent thyroid hormone at hormonogenic site B is accompanied by a substantial alteration of neighboring structural elements as compared with pre-hormonogenic human TG^6^ (Fig. 4a, c). This region, formed by the folding of four TG segments comprising 81 amino acid residues, is 92.6% identical and 97.5% similar to human TG (Table 2); the non-conserved residues occupy peripheral positions (Fig. 4c) and are thus unlikely to play a significant role in the structural differences. The observed conformational alterations are therefore far more likely to reflect the process of hormone formation than any inherent structural divergence of human and bovine TG in this region.

**Table 2:** Sequence comparison of human and bovine TG in the region encompassing hormonogenic site B.

Hormonogenic site-B is formed by the folding of four TG segments:
Segment 1: identity 88.2% and similarity 100% over 17 residues:
human 668 MQSLMGSQPAGSTLFVP 684
bovine 668 MQSLLGSQPAGSSLFVP 684
Segment 2: identity 100% and similarity 100% over 10 residues:
human 2467 TKLLAVSGPF 2476
bovine 2467 TKLLAVSGPF 2476
Segment 3: identity 96.9% and similarity 100% over 32 residues:
human 2515 GLINRAKAVKQFEESRGRTSSKTAFYQALQNS 2547
bovine 2515 GLINRAKAVKQFEESQGRTSSKTAFYQALQNS 2547
Segment 4: identity 86.4% similarity 90.9% over 22 residues:
human 2563 WYYSLEHSTDDYASFSRALENA 2584
bovine 2563 WYYSLEHDSDDYASFSRALEQA 2584
Overall site-B: identity 92.6% and similarity 97.5% over 81 residues
Overall TG conservation: identity 77.4% and similarity 85.9% over 2771 residues

The region is formed by four segments (Segments 1-4) comprising a total of 81 amino acid residues. Conservation was measured on the MBOSS-matcher using a BLOSUM62 evolution matrix.

A comparison with the local structure of site B in pre-hormonogenic human TG (Fig. 4a, c) reveals discrete rearrangements with a potential bearing on hormone formation and/or stabilization. The helix-loop-helix motif formed by residues 2515–2548 (dark gray-blue) is substantially reorganized, rotating F2539 (green) by ~80° and displacing it by ~9 Å from its initial position. This rearrangement moves the acceptor tyrosine (red) away from its close apposition to F2576 (gold), flips the residual donor site far away, and packs the double-ringed reaction product into an interior pocket bounded by α-helices. The large change in position of F2539 potentially stabilizes the nascent hormone by CH-Pi stacking with both F2576 (now liberated from its apparent interaction with the acceptor tyrosine) and the aromatic ring of the acceptor tyrosine (~3.5 to 4 Å distance in each case). In addition, a loop-helix motif composed of residues 2566-2585 (pale red) and a loop composed of residues 2471-2475 (pale green) reorganize to stabilize or accommodate the configuration changes. This local reorganization does not significantly impact the overall structure of TG.

## Discussion

The results presented here provide a structural view of endogenous thyroid hormone formation and establish a context for its mechanistic investigation at the atomic scale. It is probable that at least some of the local TG conformational rearrangements accompanying the presence of nascent thyroid hormone at site B support the oxidative coupling of the hormonogenic tyrosine residues and the transfer of the phenolic ring from donor to acceptor. In the pre-hormonogenic conformation of human TG, the respective hormonogenic sites A-D do not appear to exhibit conserved local structural features^6^, but it is possible that common features appear at the sites with the local structural reorganization that likely accompanies the formation of the nascent hormone. One possibility for a common mechanistic theme is that local rearrangements bring aromatic side chains into position to favor hormone formation or stabilization at other hormonogenic sites, similar to what we suggested for F2539 and F2576 at site B. Biochemical experiments indicate that the structural requirements for the tyrosine coupling reaction to take place are not especially strict^6^, so it is also plausible that different catalytic and stabilization mechanisms involving disparate structural elements will be involved at some or all of sites A, C, and D. A deeper understanding of the mechanism will require a comparison of the structure reported here with structures of TG with nascent thyroid hormone present at the other hormonogenic sites.

While this work was undergoing peer review, a paper was published describing a cryo-EM structure of native bovine TG with nascent thyroid hormone present at hormonogenic sites A and B^9^. The structural organization of the region encompassing site B is essentially identical to that reported here. The structural rearrangement described at site A appears to have little in common with that of site B, suggesting diverse structural underpinnings of thyroid hormone formation and stabilization.

It is possible that at least some of the structural reorganization accompanying the presence of thyroid hormone at site B serves additional functions of TG^1–3^, although this possibility need not exclude a role in the hormone formation mechanism. For example, the positions of pre-hormonogenic acceptor Y2573 and donor Y2540 could reflect an initial requirement that the tyrosine residues be accessible to solvent to permit iodination, whereas the packing of the newly-formed thyroid hormone into a deeper interior pocket could reflect a subsequent requirement that it be shielded from solvent for storage and trafficking prior to TG proteolysis and hormone secretion.

## Methods

### Streptavidin-affinity grid preparation

Copper R2/1 or R2/1 holey carbon grids (Quantifoil/C-Flat) were washed overnight in chloroform vapor and then coated with a ~10-nm layer of carbon by evaporation. The next day, grids were covered with liquid hexane for 10 minutes^10^. Hexane was removed by pipette, and grids were dried for 90 mins. A biotinylated lipid monolayer was established^8^ using a solution of 1 mg/ml of 16:0 biotinyl-PE (870285 P, Avanti Polar) dissolved in a mixture of 65:35:8 chloroform/methanol/water. Unlike the protocol in ref. ^8^, we omitted spreading talcum powder onto a castor oil surface before the addition of biotinylated lipids. The lipid monolayer was transferred onto grids by briefly touching the layer of lipid tails with the carbon side of the grid. Unbound lipid was then washed out by touching the grid sequentially to three 50-μL drops of subphase buffer^8^. Excess buffer was removed by touching a filter paper to the edge of the grid. Immediately after blotting, grids were loaded carbon-side-down onto ice-cold 10 µl drops of streptavidin solution at 0.15 mg/ml (N7021S, NEB) in a 24-well sitting-drop crystallization plate (Cryschem, Hampton Research), which must be at room temperature. Transfer of the lipid monolayer and washing of grids were performed as described^8^. Humidity was controlled by filling the reservoirs with 1 ml double-distilled water (room temperature) and by sealing the wells with grease and siliconized cover slides. Crystallization of streptavidin proceeded for 25–30 minutes at 20–22 °C. Grids were washed twice by adding and removing 100 µl of wash buffer between the grids and the pedestal by pipette. After the second wash, 100 µl of buffer was added and the grids were lifted from the drops using anti-capillary tweezers. Excess buffer was blotted off and grids were dried with the carbon-coated-side-up on a filter paper for 40 minutes. As described^8^, crystals were stabilized by depositing 1–2 nm of carbon by evaporation on lipid tails. We obtained optimal results by limiting the number of times carbon was deposited to fewer than nine.

### Biotinylation and purification of Thyroglobulin (TG)

We purchased *Bos taurus* endogenous TG from Sigma (T9145). TG was dissolved in TG buffer (20 mM HEPES pH 7.4, 150 mM NaCl) to a concentration of 5 mg/ml. Solution was clarified by centrifugation for 20 minutes at 21,000 x *g*, 4 °C. Biotinylation reagent (NHS-[PEG]_12_-biotin, EZ-Link A35389) was dissolved in TG buffer to a concentration of 5 mM. Biotinylation was performed on 200 µl of TG solution using a 5-, 10-, or 20-fold molar excess of biotinylation reagent. Biotinylation proceeded for 30 min at room temperature and was terminated by quenching on ice with 5 mM Tris pH 7.4. Unreacted biotinylation reagent was removed from the sample by gel filtration on a Superose-6 Increase 10/300 GL column. The fraction corresponding to the peak TG concentration was used for the next steps. To analyze the results of the reactions with differing biotinylation ratios, we performed a sample capture using streptavidin beads^11^ (Thermo Scientific 20357). 4 µl of streptavidin bead slurry (50%) was placed in 1.5-ml disposable tubes and washed twice for 5 min with 500 µl of Tg buffer +/− 1 mM biotin. Beads were equilibrated for 30 minutes at 4 °C on a head-over-head agitator with 500 µl of TG buffer +/− 1 mM biotin. Beads were pelleted at 500 x g for 1 min at 4 °C, and excess supernatant was discarded. Tubes were weighed again, and buffer was added or removed to balance their masses. 2 µl of biotinylated sample were added to the tubes and incubated for 30 min at 4 °C on a thermomixer (950 rpm). Beads were pelleted at 500 x g and 4 °C, and 10 µl of supernatant was then transferred into chilled PCR tubes. 0.5 µl were analyzed by dot-blot with Streptavidin-HRP (SA10001, ThermoFisher Scientific) and 9.5 µl were analyzed by SDS-PAGE, stained with the silver nitrate method (Invitrogen). Biotinylated TG from the 10-fold molar excess biotin reaction was selected for cryo-EM analysis because it showed approximately 5% residual un-biotinylated TG, suggesting that most of the biotinylated TG molecules in the sample carried only one or a few biotins.

### Liquid chromatography-tandem mass spectrometry (LC-MS/MS) analysis

Bovine TG (10 µg) was loaded onto a denaturing 4-12% Bis-Tris NuPAGE gel (Invitrogen) and electrophoresed at 150 V for 1 h at room temperature. The TG band was fixed with 20% ethanol/10% acetic acid, visualized with QC colloidal Coomassie stain (BioRad), and destained with nanopure water. A slice containing the TG band (330 kDa) was cut from the gel for LC-MS/MS analysis at the Harvard Medical School Taplin Biological Mass Spectrometry Facility using an Orbitrap Exploris 480 (Thermo Scientific) coupled to an ultra-HPLC pump. TG was denatured and digested with trypsin and chymotrypsin. Analytical LC separations were performed on 100 µm ID silica columns packed with Accucore C18 (2.6 µm, Thermo Scientific). Assignment of MS/MS spectra was performed using the SEQUEST algorithm (ver. 28, rev. 13) utilizing the FASTA sequence for bovine thyroglobulin (UniProt ID P01267). SEQUEST searches were performed while requiring peptide termini to have trypsin and chymotrypsin specificity. Carbamidomethylation of cysteine residues (+ 57.0215) was set as a static modification. The following were set as dynamic modifications: oxidation of methionine residues (+15.9949), tyrosine di-iodination (+ 251.793), T3 from tyrosine (+ 469.7161), and T4 from tyrosine (+ 595.6128).

### Cryo-EM sample preparation

Streptavidin-affinity grids were rehydrated and equilibrated with TG buffer as described^8^. Excess TG buffer was blotted off and grids were incubated with 4 µl of biotinylated TG sample for 20 minutes at room temperature in a high-humidity chamber made up of a specimen transport box (Nalgene C1812) containing a wet filter paper. During incubation, the tweezers carrying the grids rested on an ice-cooled layer of aluminum. Unbound TG was removed by washing consecutively with three 50-µl drops of TG buffer. The concentration of biotinylated TG used for the preparation of frozen-hydrated samples was estimated by negative stain electron microscopy (we found that apparent particle densities for a biotinylated sample on streptavidin affinity grids were similar in negative stain and cryo-EM analysis). For room temperature imaging, grids were negatively stained with 2% uranyl acetate for 2 min. For frozen-hydrated specimens, grids were processed as described^8^ using a Vitrobot Mark IV (ThermoFisher Scientific) and stored in liquid nitrogen until imaging.

### EM data collection

Room temperature imaging was performed on a Tecnai G_2_ Spirit BioTWIN equipped with an AMT 2k CCD detector, operated at 120 keV at a nominal magnification of 36,000 (3.6 Å/pixel). Cryo-EM data collection was performed on a Titan Krios (ThermoFisher Scientific) transmission electron microscope at a magnification of 105,000X, operated at 300 keV, and equipped with a GIF Quantum energy filter (slit width, 20 eV, Gatan) and a K3 direct electron detector (Gatan). We used Serial-EM^12^ for automated data collection using beam-image shift, with four target holes per stage movement and four recordings per hole. 34,597 movies were recorded in counting mode, using a dose of 53.7 7 e.A^−2^ (dataset 1) and 54.15 e.A^−2^ (dataset 2) with a sampling of 50 frames per movie at a pixel size of 0.825 Å.pix^−1^.

### Data processing

Movies were imported into Relion-3.1^13,14^, and beam-induced motion correction and dose-weighting were performed using MotionCor2^15^. Datasets 1 and 2 were imported as two independent Relion optic groups. The streptavidin crystal lattice was filtered out from micrographs using a MATLAB script kindly provided by the laboratory of R. M. Glaeser (see Ref. ^8^). Contrast transfer function parameters were estimated with CTFFIND-4^16^. 6,749,503 particles were picked with CryOLO^17^ using a model trained on streptavidin-affinity grids. Particles were extracted in Relion in boxes of 512 pixels, binned to 128 pixels. Particles that were damaged or incomplete, particles in locations where lattice subtraction was faulty, and contaminants were discarded by two rounds of 2D classification. Only classes with robust secondary structure features were selected, resulting in a dataset of 2,534,214 particles.

An ab-initio model was generated without symmetry, then aligned to the C2 axis and symmetrized. 3D classification with C2 symmetry was performed to get rid of particles not yielding to classes with secondary structure features, resulting in a dataset of 732,593 particles. A consensus 3D refinement with C2 symmetry was performed from which particles were symmetry-expanded. We then performed focused 3D classification in C1, without alignment, using a mask surrounding one asymmetric unit (ASU). Particles belonging to classes in which a complete N-terminal domain was visualized^6^ (Supplementary Fig. 1) were selected (890,332). Particles were re-extracted with re-centering in a box of 512 pixels, without binning.

Processing for the ASU map: Focused refinement was performed using the 890,332 un-binned, C2-expanded particles, leading to a nominal resolution of 3.43 Å based on the FSC 0.143 criterion. To reduce heterogeneity, a local search was performed with 5 classes. 588,070 particles from the 3 best classes were used for an additional step of focused refinement, leading to a resolution of 3.46 Å based on FSC with criterion 0.143. Bayesian polishing was performed. While this step reintroduced the streptavidin crystal lattice, Bayesian polishing did not impair downstream particle alignment. CTF refinement was also performed, but no map improvement was observed. Polished particles were loaded in cryoSPARC^18^ for local refinement using the non-uniform regularization protocol^19^. This procedure yielded a map with a resolution of 3.35 Å based on FSC (0.143 criterion). We improved interpretability of the map using density modification^20^ in Phenix^21^.

Processing for the C2 map: 890,332 C2-expanded particles described above underwent alignment-free, focused classification with a mask on the adjacent ASU. 340,508 particles from 1 class with a complete asymmetric unit were selected, after which duplicates generated by symmetry expansion were removed. This procedure resulted in a final subset of 242,813 particles in which both asymmetric units, including the N-terminal domains, were fully visualized, corresponding to fewer than 10% of the initial number of particles. We performed 3D refinement with C2 symmetry, leading to a ~3.6 Å map. Particles were polished, loaded in cryoSPARC, and further refined using the non-uniform regularization protocol, yielding a 3.28 Å map. To improve the map quality for flexible elements around the C2 axis, particles were C2-expanded and locally refined using non-uniform refinement. This procedure produced two additional maps, one for each ASU, at 3.26 Å and 3.27 Å. C2 and ASU maps were combined in Phenix to generate the final ~3.3 Å full homodimer map.

Model construction was performed using the C2 combined map in Coot^22^ (CCP4 suite), and model refinement was carried out in Phenix 1.19 using real-space refinement with secondary structure restraints and Ramachandran restraints. A density-modified, single-ASU map was used for better modeling and interpretation of hormonogenic site B.

### Reporting summary

Further information on research design is available in the Nature Research Reporting Summary linked to this article.

## Supplementary information

Supplementary Information

Reporting Summary

## Data Availability

Datasets, maps and models generated in this study have been deposited at the RCSB Protein Data Bank (PDB) (https://www.rcsb.org/) under accession code 7QTQ and at the Electron Microscopy Data Bank (EMDB) under accession code EMD-14145. Source data are provided with this paper.

## Source data

Source Data
