# Supplementary material for: Formation of thyroid hormone revealed by a cryo-EM structure of native bovine thyroglobulin
Source: Nat Commun. 2022 May 2;13:2380. doi: 10.1038/s41467-022-30082-4 (PMC9061844; doi:10.1038/s41467-022-30082-4)
Supplement: Supplementary file 1 — Supplementary Information [file 41467_2022_30082_MOESM1_ESM.pdf]

Supplementary Information

Formation of thyroid hormone revealed by a cryo-EM structure of native bovine thyroglobulin

Nils Marechal, Banyuhay P. Serrano, Xinyan Zhang, Charles J. Weitz

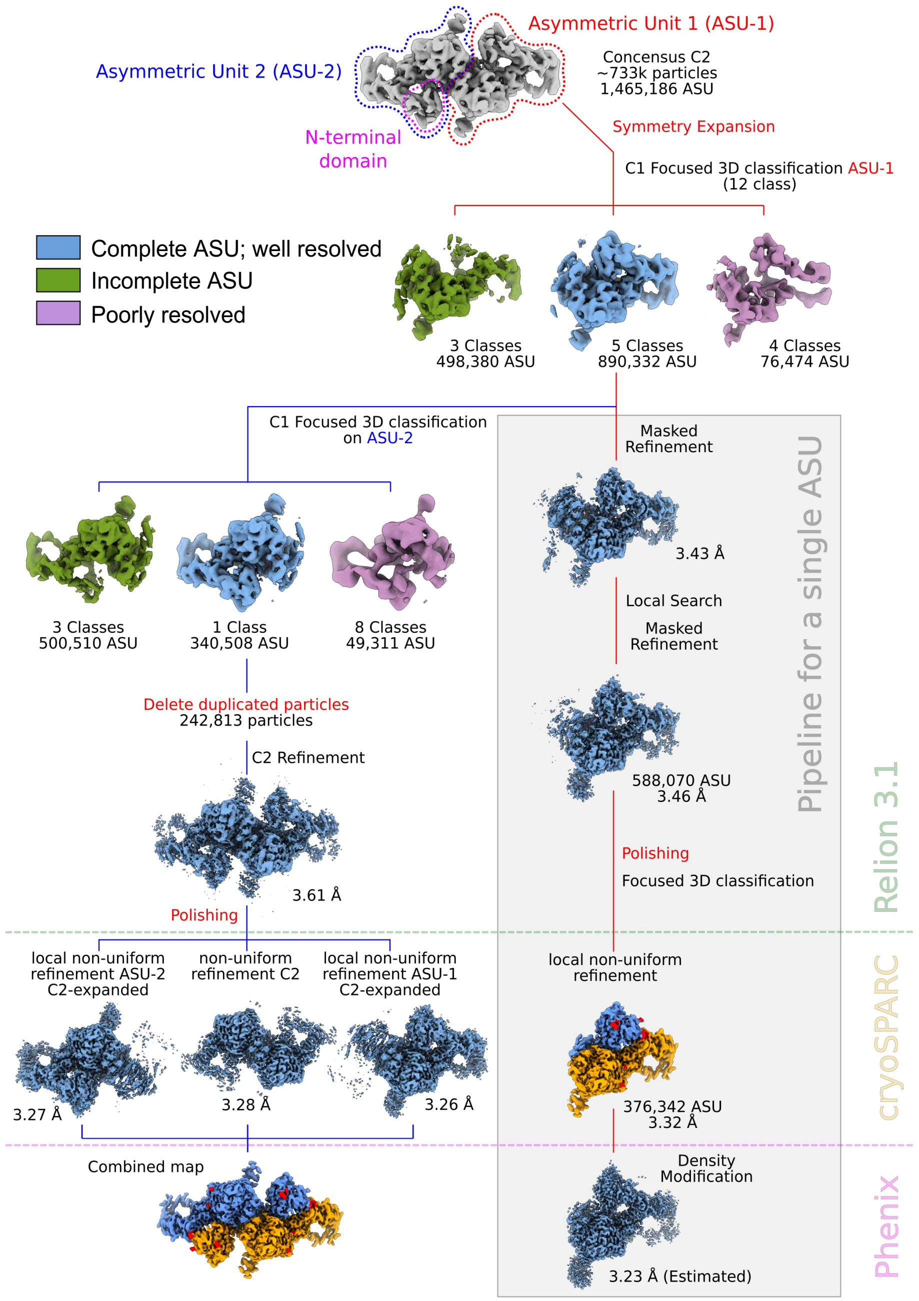

Supplementary Fig. 1. Scheme for 3D classification and refinement of native bovine TG. The gray map at the top is the C2 consensus model obtained after pooling datasets 1 and 2 prior to exclusion of particles without a folded N-terminal domain (pink dashed circle). The quality of each class is color-coded according to the legend at upper left. The left wing of the figure shows steps of data processing performed to calculate the C2 final model. The right wing (light gray panel) shows steps of data processing performed to calculate the asymmetric unit (ASU) map used for solving structural details of hormonogenic site B.

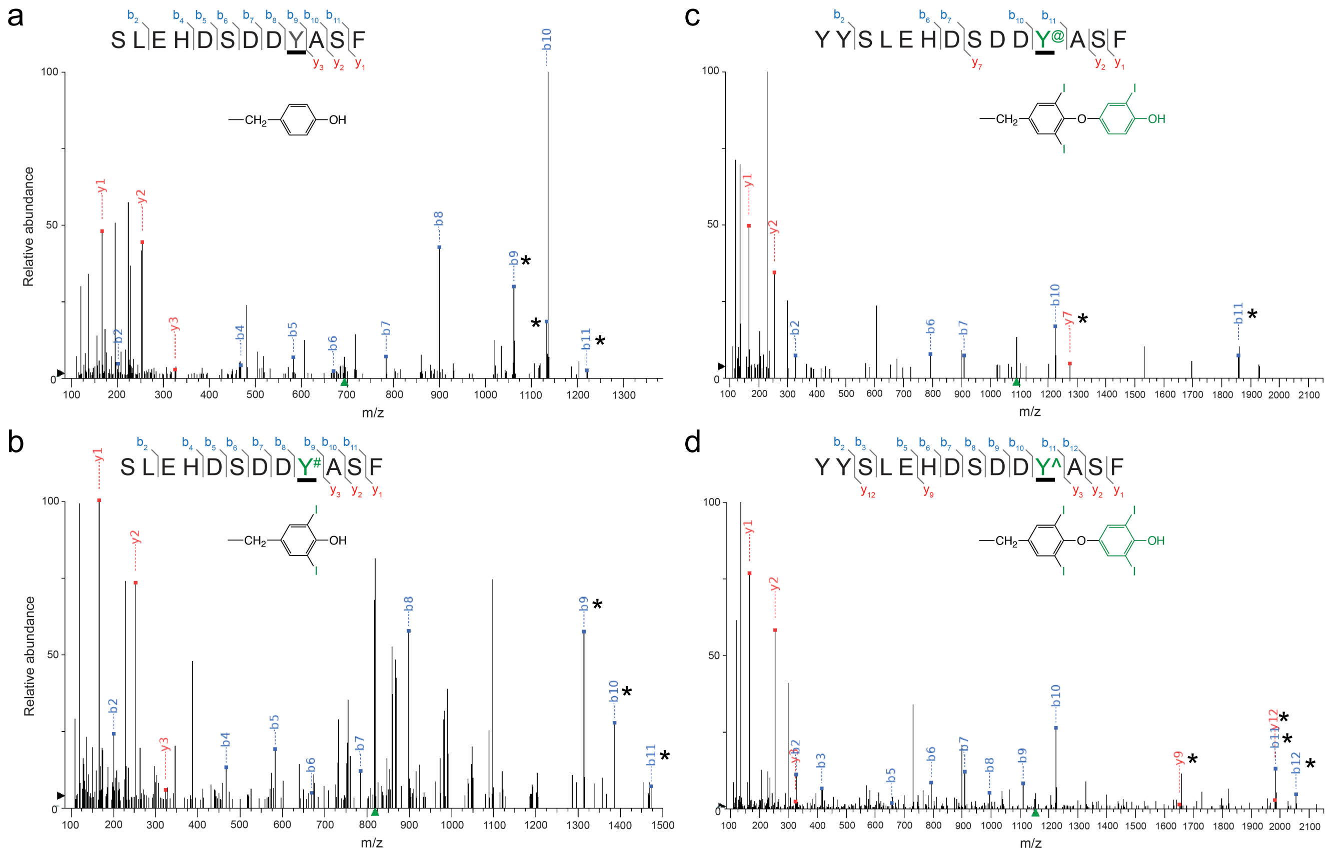

Supplementary Fig. 2. Tandem mass spectrometry analysis of Y2573 modification (hormonogenic site B) in sample of native bovine TG. Each spectrum shows the fragments generated from the TG peptide at the top of the panel (Y2573 is underlined). $b_{1\text{-}n}$, N-terminal fragments. $y_{1\text{-}n}$, C-terminal fragments. Green triangle on X-axis indicates the precursor ion. Asterisks, peaks indicating the mass addition corresponding to the Y2573 structure shown in the panel. **a**, Unmodified Y2573. **b**, di-iodinated Y2573; mass addition: 251.7933. **c**, nascent T3; mass addition: 469.7161. **d**, nascent T4; mass addition: 595.6128.

N-linked glycosylation sites

	Residue
Confirmed sites:	2250
	2122
	495
	483
	198
	2014
	2295
Possible sites:	2456
	1365

Supplementary Table 1. N-linked glycosylation sites in native bovine TG.
